# Contact Resistance Modeling Under Complex Wear Conditions Based on Fractal Theory

**DOI:** 10.3390/ma18133060

**Published:** 2025-06-27

**Authors:** Changgeng Zhang, Xiaoxiao Liu, Liang Jin, Rongge Yan, Qingxin Yang

**Affiliations:** 1 State Key Lab of Intelligent Power Distribution Equipment and System, College of Electrical Engineering, Hebei University of Technology, Tianjin 300401, China; 202321401103@stu.hebut.edu.cn (X.L.); jinliang_email@163.com (L.J.); yanrg@hebut.edu.cn (R.Y.); yangqqxin@hebut.edu.cn (Q.Y.); 2 Hebei Key Laboratory of Equipment and Technology Demonstration of Flexible DC Transmission, College of Electrical Engineering, Hebei University of Technology, Tianjin 300401, China

**Keywords:** rail wear, contact resistance, fractal theory, surface profile

## Abstract

The muzzle velocity of electromagnetic rail launchers approaches 1550 m/s, exhibiting typical hypervelocity electrical contact characteristics. During the electromagnetic launching process, extreme conditions, such as high current density, high temperature rise, and strong strain can cause wear on the surfaces of the armature and rail. Electromagnetic launch tests are conducted to study the wear conditions of the rail surface and the relationship between the wear state and contact resistance. After the rail is abraded by hundreds of launching armatures, its surface 2D profile and morphological characteristics are measured and analyzed. Based on fractal theory, the static contact resistance model is developed. Concurrently, the contact resistance at various positions is measured to reveal the evolution of the static contact resistance between the armature and the rail under wear. The research results show that along the direction of the armature launch, the rail surface wear transitions from mechanical wear to electrical wear, the fluctuation range of the 2D profile becomes smoother, and the roughness of the rail surface shows a decreasing trend. When the roughness is greater, the contact resistance is more sensitive to changes in external load.

## 1. Introduction

Electromagnetic launchers have received significant attention because of their high launch kinetic energy, high energy efficiency, precise controllability, and extended range [1,2]. However, friction and wear are inevitable [3,4,5,6,7,8]. The coupling effects of electricity, magnetism, force, and heat cause composite mechanical and electrical wear on the contact surfaces of the armature and rail [9]. The influence of temperature on electrical resistivity varies across different materials. Thomas et al. investigated ceramic materials and found that their resistivity decreases with increasing temperature; however, the sensitivity of this change depends on the ceramic’s specific composition [10]. Kamila et al. reported a similar trend for nanocomposites, where the resistivity decreased as the temperature increased to 180 °C. Notably, the sensitivity of the resistivity to temperature changes was closely related to the release of free water from the pores of the cement matrix [11,12]. Liu et al. demonstrated that lubricant viscosity significantly affects the shear force, fluctuation duration, and friction-induced vibration during the initial stage of friction. In particular, low-viscosity lubricants are more effective in reducing the initial friction force and system fluctuations under low-temperature conditions, thereby lowering the coefficient of friction and reducing wear [12]. The contact characteristics of ultra-high-velocity sliding contact affect the reliability and stability of the operation of the equipment [13]. The wear behavior of the rail surface influences the contact characteristics between the armature and rail, affecting launch efficiency and reducing rail lifespan [14].

Currently, research on rail wear primarily focuses on numerical simulations and experimentation [15,16,17,18,19]. To study the internal damage mechanisms during electromagnetic launch, Dong et al. developed an internal profile detector. By comparing the measured actual surface profile with the ideal surface profile, they obtained the dam-age profile. Two- and three-dimensional profile graphs are plotted to visually observe the extent of damage, providing more precise data for damage analysis [20]. Gao et al., to study the effects of electromagnetic force and preload on the contact characteristics and wear of the armature-rail interface, used ANSYS to establish a three-dimensional simulation model based on the Archard model. They determined the impact of the armature’s interference and acceleration on wear [21]. Ren et al. used HyperMesh and ANSYS to predict wear based on the Archard wear model and found that the wear between the armature and rail is proportional to the load. Additionally, wear is significantly affected by the friction coefficient, which can be reduced by improving lubrication conditions [22].

Currently, the calculation of the contact resistance between the armature and rail mainly relies on numerical simulations and experiments to obtain the muzzle voltage, as well as laboratory static measurements to determine the static contact resistance between the armature and rail [23,24,25,26,27]. Zhu et al. established a numerical model for contact resistance and friction coefficient between the armature and rail. By analyzing the muzzle voltage, pulse current, and armature displacement velocity curves collected from electromagnetic launch tests, they calculated the contact resistance and friction coefficient, thereby verifying the accuracy of the model. They also analyzed the impact of rail surface wear on the reliability of the sliding contact between the armature and rail [28]. Ge et al., based on the kinematics and electromagnetics of electromagnetic launch, eliminated the influence of induced electromotive force in muzzle voltage on contact resistance. They established a contact resistance model, revealing the relationships among the contact resistance, muzzle voltage, armature velocity, and pulse current, and analyzed the contact state between the armature and rail during the actual launch process [29].

Most current research studies the wear mechanisms of rail and armature and the characterization of contact resistance separately, with limited research focusing on the impact of rail surface damage on contact resistance. To address this issue, this study scanned the rail after electromagnetic launch to observe the damage morphology of the rail surface at different locations. By extracting the two-dimensional profile curves of the rail surface and measuring the static contact resistance at different positions using a resistance measuring instrument, we explored the relationship between rail surface wear and static contact resistance under different external loads.

## 2. Experimental Methods

### 2.1. Experimental Materials

The rail material is alloy steel, with rail length of 2.1 m. The composition and heat treatment process of the alloy steel were provided by the manufacturer. The main chemicals of alloy steel are shown in Table 1. The heat treatment process involved austenitizing the alloy steel at 850 °C for 30 min, followed by rapid oil quenching at 60 °C, tempering at 560 °C for 1.5 h, and final air cooling to room temperature. The armature material is aluminum alloy. The alloy steel has good electrical conductivity and strong resistance to plastic deformation, while the aluminum alloy has a low density and high strength. The main properties of the armature and rail materials are listed in Table 2.

### 2.2. Experimental Set-Up

**Figure 1:**
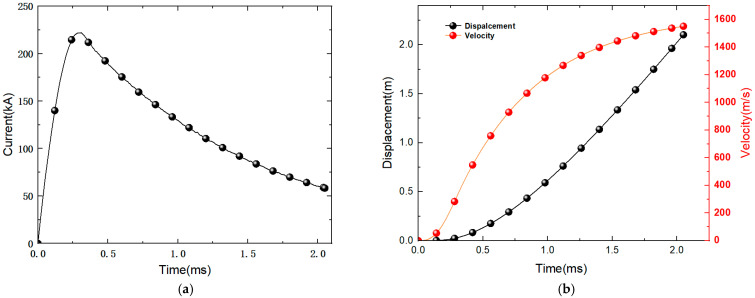
The curves of launching current and the velocity and displacement of the armature: (**a**) The launching current; (**b**) Velocity and displacement of the armature.

After multiple repeated launches, the rail is separated from the launching system. The current during the process is given in Figure 1a,b, which shows the velocity and displacement vs time graph of the armature. The maximum peak value of the current is 220 kA, and the armature muzzle velocity is about 1550 m/s. Based on the armature’s velocity and displacement vs time graph during the launch process, the rail is segmented along the direction of the armature launch. The rail position corresponding to the launch time of 0–0.4 ms is considered the low-velocity stage, the position corresponding to 0.4–1.5 ms is considered the acceleration stage, and the position corresponding to 1.5–2 ms is considered the high-velocity stage.

**Figure 2:**
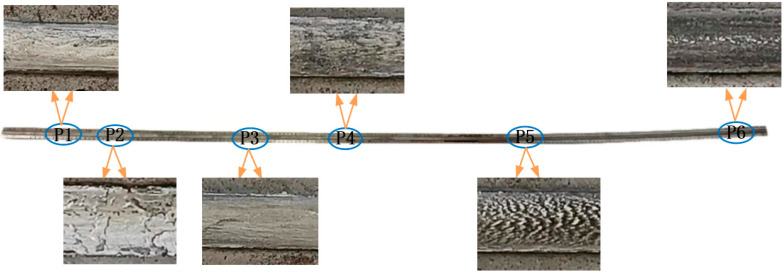
Selected position and morphology of the rail.

Based on the typicality of the morphological characteristics, different positions along the direction of armature launch are used for morphological observations at different stages of the rail. The selected positions and the morphologies of the surfaces are shown in Figure 2. Positions P1 and P2 correspond to the low-velocity stage, positions P3 and P4 correspond to the acceleration stage, and positions P5 and P6 correspond to the high-velocity stage. From the morphological images of the surface, it can be observed that along the direction of armature launch, the deposition layer covered almost the entire rail surface, and the color of the deposition layer gradually changed from silver-gray to gray-black.

### 2.3. Surface Morphology Set-Up

In this study, a scanning microscope method is used to observe the damage morphology of the rail surface and extract two-dimensional profile lines. For this purpose, an LCubor AST non-contact industrial 3D camera is used, which has a vertical resolution of 1 μm and a central field of view of 36 mm × 27 mm, allowing for effective profile extraction over a larger area of the rail.

### 2.4. Electrical Contact Resistance

**Figure 3:**
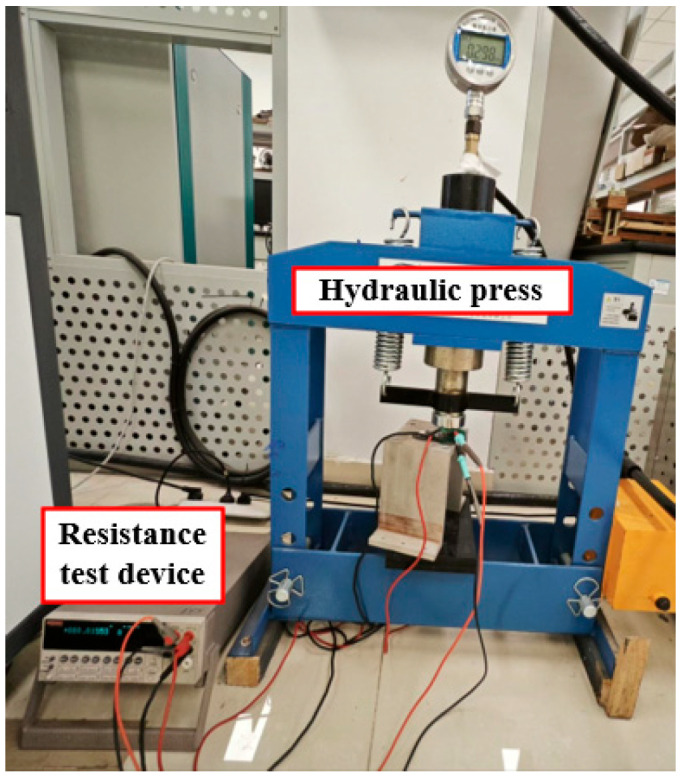
Static contact resistance measuring set-up.

To measure the static contact resistance between the rail and armature, a measurement set-up is designed, consisting of a press supply device and a resistance measurement device, as shown in Figure 3. The external load supply device used a hydraulic press that can provide a maximum external load of 2 × 10^5^ N. The resistance test device is a KEITHLEY 2002 digital multimeter (Keithley Instruments, Solon, OH, USA), which has a wide measurement range and high accuracy. The static contact resistance is measured using the four-terminal method to enhance measurement accuracy.

## 3. Experimental Results

### 3.1. Observation of Surface Morphology

**Figure 4:**
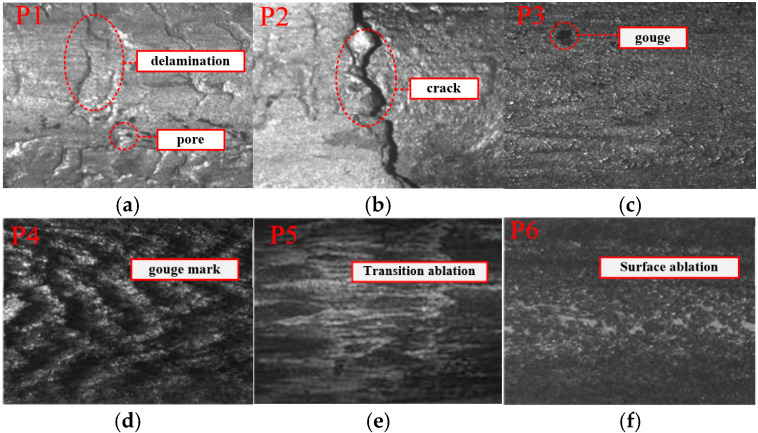
Microscopic morphology of the rail surface: (**a**) The position of P1; (**b**) The position of P2; (**c**) The position of P3; (**d**) The position of P4; (**e**) The position of P5; (**f**) The position of P6.

In the direction of the armature launch, positions P1 and P2 are considered the low-velocity stage, positions P3 and P4 are considered the acceleration stage, and positions P5 and P6 are considered the high-velocity stage for microscopic observation. Figure 4 displays the microscopic images of several locations. During the low-velocity launch stage, owing to the relatively low velocity of the armature movement, a significant amount of thermal energy is concentrated at the interface between the rail and the armature. As a result, melting occurred at the armature–rail contact surface. In this stage, rail damage primarily manifests as delamination of the deposited layer, pores, and cracks. Owing to the relative motion between the armature and rail, there is also some mechanical wear.

During the acceleration stage of launch, the velocity of the armature increases, the relative time for the armature to stay on the surface of the rail decreases, and the melting tendency of the rail and armature decreases. Owing to the relatively high relative velocity between the armature and rail, even a slight instability can cause a large impact force on the rail, resulting in plastic deformation of the rail.

During the high-velocity launch stage, ablation occurs in the latter half of the rail. When the armature exits the barrel, muzzle ablation occurs, leading to ablation and carbonization of the rail surface material. Carbonized particles adhere to the rail surface, causing it to blacken.

### 3.2. Surface Profile Micromorphology Measurement

**Figure 5:**
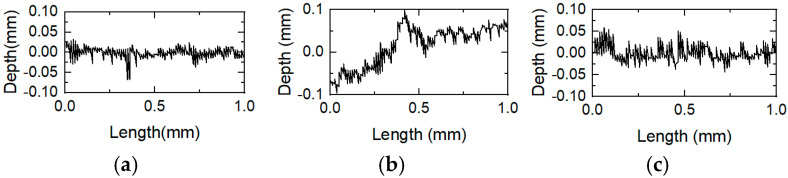

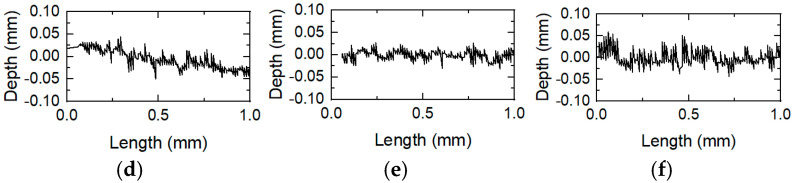
Two-dimensional profile lines at different positions of the rail. (**a**)The position of P1; (**b**) The position of P2; (**c**) The position of P3; (**d**) The position of P4; (**e**) The position of P5; (**f**) The position of P6.

During the armature’s launch along the rail, mechanical wear caused by the relative motion between the armature and the rail, frictional heat generated by the high-velocity motion between the armature and the rail, as well as Joule heating caused by contact resistance between them, lead to rough surface on the rail. Quantitative representation of the two-dimensional profile lines of the rail surface enables a more comprehensive analysis of wear conditions during electromagnetic launch processes. To further analyze rail damage at various positions along the armature launch direction, two-dimensional profile lines are measured at P1, P2, P3, P4, P5, and P6. Figure 5 shows the profile lines at different positions.

It can be seen from Figure 5 that the surface fluctuation range is the largest at position P2 and the smallest at position P6. To sum up, where the armature speed is higher, the surface fluctuation is smoother.

At position P1, the relative motion velocity between the armature and the rail is relatively low, and the Joule heat accumulation between the armature rail produces a large amount of heat energy. The armature melts, and the aluminum alloys are attached to the rail to form a deposited layer. From the above wear analysis of the rail, it can be seen that the delamination of the deposited layer and pores resulted in a rougher rail surface and large fluctuation range on the rail surface. At position P2, cracks appeared in the deposited layer on the rail surface. As can be seen from Figure 4, the cracks are relatively deep and exhibit a zigzag shape, which results in greater fluctuation range in the two-dimensional profile lines on the surface. At position P3, the relative motion velocity between the armature and the rail increased. Owing to the relatively high relative velocity between the armature and rail, a slight instability at this time will also cause a large impact force on the rail, resulting in plastic deformation of the rail surface and mechanical wear. At the same time, the relatively high velocity between the armature and rail made the rail surface smoother. The two-dimensional surface profile fluctuation range at position P3 is smaller than those at positions P1 and P2. With the increase of the relative velocity between the armature and rail, the surface profile fluctuation range of the rail decreased. At position P6, where the relative velocity between the armature and the rail is the highest, the two-dimensional profile line fluctuation range on the rail surface is the smallest.

### 3.3. Characteristics of Rail Surface Roughness

In the field of tribology, surface characteristics are currently described using four main methods: statistical methods, spectral methods, fractal methods, and morphological methods. To further study the influence of rail wear on the static electric contact characteristics of the armature rail, the statistical methods are used to describe the surface characteristics, which are represented by *R_a_* at various positions on the rail. The structure function method is used to calculate the fractal dimension *D* and characteristic scale parameter *G* of the rail surface. These parameters are used to calculate the contact resistance by substituting them into the equivalent contact resistance model. *R_a_* represents roughness.

(1) $$R_{a}=\frac{1}{l}(\int_{0}^{l}\left|z(x)\right|\mathrm{dx})=\frac{1}{N}\sum_{i=1}^{N}\left|z_{i}\right|$$

where *l* is the sampling height, *z*(*x*) is the height deviation of the surface profile, *N* is the total number of sampling points, and *z_i_* is the height deviation of the *i*-th sampling point.

**Figure 6:**
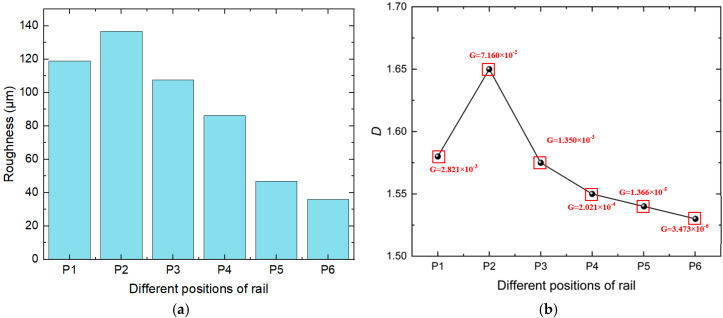
Roughness and *D* and *G* parameters at different positions of the rail: (**a**) Roughness of different positions; (**b**) *D* and *G* of different positions.

By using the height data *z*(*x*) substituting it into Equation (1), the roughness at different positions of the rail is obtained, as shown in Figure 6a, which is consistent with the two-dimensional profile fluctuations of the rail. The overall roughness decreased along the direction of the armature launch. The roughness at position P2 is the largest, because of cracks in the deposited layer at this position, which causes the surface to become rougher. By using the structure function method, the *D* and *G* are obtained in Figure 6b.

The structure function method is an effective method to calculate the fractal dimension of surface. For a surface profile *z*(*x*), its structure function is defined as:

(2) $$S(\tau )=\langle {\left[z(x+\tau )-z(x)\right]}^{2}\rangle =\int_{-\infty }^{+\infty }S(\omega )(e^{j\omega \tau }-1)d\omega =\frac{1}{N-1}\sum_{n=0}^{N-n}(z_{i+n}-z_{i}{)}^{2}=c\tau^{4-2D}$$

where *τ* = *n*_0_Δ*t*, *n*_0_ represents the number of sampling points, Δ*t* represents the sampling interval, with a total of *N* points collected, *n* is the sequence number, and *c* is a constant related to the fractal dimension *D* of the profile and the characteristic scale parameter *G*. For *τ* on different scales, calculate the structure function *S*(*τ*), by performing a linear fit of lg *S*(*τ*) versus lg *τ*. The slope *K* of the resulting line is obtained. The fractal dimension *D* is given by:

(3) D=(4−K)/2

(4) $$c=G^{2(D-1)}\frac{\Gamma (2D-3)\sin\left[\pi \left(2D-3\right)/2\right]}{2-D}$$

where Γ(*x*) is the gamma function and lg *c* is the intercept of the fitted lg *S*(*τ*) and lg*τ* lines.

### 3.4. Research on Electrical Contact Resistance

Along the launching direction of the armature, the degree of damage at each position on the rail surface is different, resulting in different fluctuations in the two-dimensional profile lines at each position. The actual contact area between the armature and the rail is different, so the damage of the rail surface affects the contact characteristics during the actual launch process. To study the influence of the surface topography at different rail positions on the contact characteristics, the contact resistance model is established based on fractal theory, considering the external load and actual contact areas. Static contact resistance measurements are conducted at six selected rail positions. Using a hydraulic press, external load is applied to the armature and rail, and the contact resistance at different positions after the external load application is measured.

#### 3.4.1. Contact Resistance Equivalent Model

**Figure 7:**
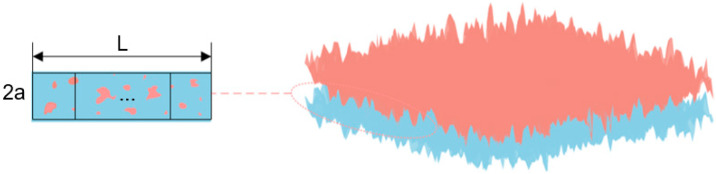
Two-dimensional profile lines at different positions of the rail.

Under the influence of external load, the armature and rail press against each other, resulting in elastic deformation. The equivalent contact surface between the armature and rail is simplified as shown in Figure 7, where 2*a* represents the width of the nominal contact surface, and *L* is the length of the nominal contact area. The area of the nominal contact surface is expressed as

(5) Aa=2aL

Holm proposed that when the current flows through the two contact surfaces, the actual contact only occurs on tiny contact spots, and the current path contracts at the contact spots, generating constriction resistance. When the materials of the two contact surfaces are different, the constriction resistance of a single conductive spot can be expressed as

(6) $$R_{s}=\frac{\rho_{1}+\rho_{2}}{4b}$$

where *ρ*_1_ is the resistivity of conductor 1 and *ρ*_2_ is the resistivity of conductor 2.

Owing to the formation of oxide film and other contaminants on the surfaces of the material, when current flows through these film layers, film resistance will occur. The film resistance and the constriction resistance are in series, so the actual contact resistance can be expressed as the sum of the film resistance and the constriction resistance. But in fact, the conductive spots are often formed under the action of contact force, and the film layer is destroyed. Therefore, the contact resistance in this study ignores the influence of film resistance.

To consider the effect of the ratio of the actual contact area to the nominal contact area on the contact resistance, it is assumed that the forces on the armature and rail are uniformly distributed and that the conductive spots are uniformly distributed across the contact surface; the nominal contact area is divided into *N* small squares with side lengths of 2*a*, as shown in Figure 7. The conductive spots in the small square are regarded as circles, and the area of the conductive spots is the actual contact area of the small square. Therefore, the actual contact resistance can be regarded as *N* parallel conductive contact resistance.

The number of small squares *N* can be expressed as

(7) N=L/2a

Assuming that the actual contact area is *A_r_*, the radius *b* of the conducting spot in the small square is

(8) $$b=\frac{2a}{\sqrt{\pi }}\sqrt{\frac{Ar}{Aa}}$$

Substituting the radius of the conducting spot into Equation (6), the contact resistance of the conducting spot in the small square can be obtained:

(9) $$Rs=\frac{\sqrt{\pi }(\rho_{1}+\rho_{2})}{8a}\sqrt{\frac{A_{a}}{A_{r}}}$$

The actual contact resistance is composed of *N* parallel conductive contact resistances and the actual contact resistance is given by

(10) $$Rc=\frac{Rs}{N}=\frac{\sqrt{\pi }(\rho_{1}+\rho_{2})}{8Na}\sqrt{\frac{A_{a}}{A_{r}}}$$

Substituting Equation (7) into Equation (10) yields

(11) $$Rc=\frac{\sqrt{\pi }(\rho_{1}+\rho_{2})}{4L}\sqrt{\frac{A_{a}}{A_{r}}}$$

Based on fractal theory, the relationship between static contact force and actual contact area is as follows:

(12) $$\left\{\begin{array}{c} F=(\frac{4\sqrt{\pi }}{3}EG^{D-1}g_{1}(D,G)+Hg_{2}(D,G))\times {A_{r}}^{D/2}(D\neq 1.5)\\ F=EG^{D-1}Ar^{4/3}\ln(\frac{Ar}{G^{2}{\left(2E/H\right)}^{2/(D-1)}})+HAr^{4/3}G^{1/2}{\left(2E/H\right)}^{1/(2D-2)}(D=1.5) \end{array}\right.$$

where the *g*_1_ and *g*_2_ can be expressed as follows:

(13) $$\left\{\begin{array}{l} g_{1}(D,G)=\frac{D}{3-2D}{\left(\frac{2-D}{D}\right)}^{\frac{D}{2}}\cdot [{\left(\frac{2-D}{D}\right)}^{\frac{3-2D}{2}}-G^{\left(3-2D\right)}{\left(\frac{2E}{H}\right)}^{\left(3-2D)/(D-1\right)}]\\ g_{2}(D,G)={\left(\frac{D}{2-D}\right)}^{\frac{2-D}{2}}\cdot G^{\left(2-D\right)}{\left(\frac{2E}{H}\right)}^{\left(2-D)/(D-1\right)}\\ \frac{1}{E}=(1-\upsilon_{c}^{2})/E_{c}+(1-\upsilon_{s}^{2})/E_{s} \end{array}\right.$$

where *E* is the effective elastic modulus, *H* is the hardness of the rough rail. *ν_c_* and *ν_s_* are the Poisson ratios of armature and rail, respectively. *E_c_* and *E_s_* are elastic modulus of armature and rail, respectively.

In Equation (12), the nonlinear relationship between the external load and the actual contact area is expressed. As the external load increases, the actual contact area also increases. In addition, the fractal dimension *D* and the characteristic scale parameter *G* also affect the actual contact area.

#### 3.4.2. Contact Resistance Model Validation and Test Results

The contact between the armature and rail is not completely smooth, but rather rough. The rough surface is composed of uneven spots, contact occurs between these asperities, and the contact resistance arises between them.

To validate the accuracy of the proposed contact resistance equivalent model, the contact resistance experimental measurements under different contact external loads are carried out at different positions of the rail. These experimental results are compared with the contact resistances calculated using the contact resistance equivalent model.

To ensure accurate contact resistance measurements, two rail segments of equal length are cut and polished using sandpaper to achieve the same surface roughness. The armature is then placed between the two rail segments, and varying external loads are applied using a hydraulic press. The contact resistance is measured under these conditions and denoted as *R*_1_. Simultaneously, the bulk resistance of the armature is measured using the four-terminal method, yielding an *R*_2_ value of 0.021 mΩ. Next, one of the original rail segments is paired with a rail segment from a different location of the rail, with the armature still positioned between them, and the contact resistance is measured under the same external load conditions and recorded as *R_3_*. Finally, the contact resistance between the armature and rail surfaces is calculated as *R_c_* = *R_3_* − (*R_1_* + *R_2_*)/2.

For the model calculations, the nominal contact area *A_a_* is 4.4149 × 10^−4^ m^2^, and *H* is taken as 150 HB. The remaining parameters are listed above.

To validate the accuracy of the model, it is compared with the GW model and experimental data. The parameters used are the same as those in the model proposed in this study.

The GW model, known as the Greenwood–Williamson model, is an elastic contact theory [30]. The calculated contact resistance values obtained from the GW model are summarized in Table 3.

**Figure 8:**
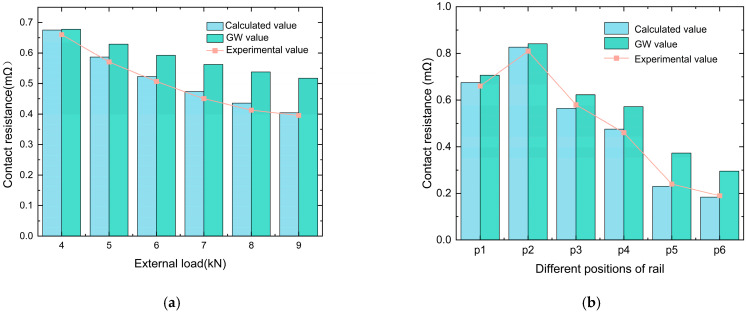
Comparison of contact resistance between experimental and calculated values: (**a**) Contact resistance at P1; (**b**) Contact resistance at 4kN.

Figure 8a compares the calculated contact resistance values of the equivalent model, GW model, and experimental measurements at position P1 under different external loads. Figure 8b presents a comparison of the calculated values of the equivalent model, GW model, and experimental measurements of contact resistance at various positions along the rail under an external load of 4 kN. It can be observed that the results from the model proposed in this study and the GW model follow the same trend as the experimental measurements. Under the same surface roughness conditions, contact resistance decreases as the external load increases; similarly, under the same contact external load, contact resistance decreases as roughness decreases. Notably, the GW model exhibited lower sensitivity to external load changes. This is because in the GW model, the radius of the contact spot is related to the external load by a cubic root relationship, making it less sensitive to external load variations, which affects its accuracy. Compared with the experimental measurements and the calculated values from the model in this study, the GW model tends to overestimate the overall contact resistance. This is because the GW model focuses on the overall surface roughness while lacking local topological information. The model proposed in this study produces results with smaller discrepancies from the experimental measurements under different external loads, with errors within acceptable limits.

**Figure 9:**
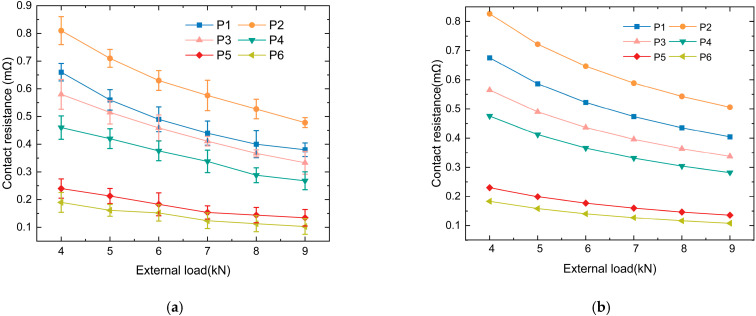
The contact resistance values obtained from experimental measurements and the model proposed in this study: (**a**) The contact resistance values in the model; (**b**) The contact resistance values in the model.

Figure 9a shows the curves of static contact resistance between the armature and rail at different positions on the rail as the contact external load varies. The results indicate that, along the armature launch direction, with constant roughness, the static contact resistance between the armature and rail decreases as the contact external load increases. This is because, with the increase in external load, more asperity comes into actual contact between the armature and the rail, leading to an increase in the actual contact area and a decrease in contact resistance.

At different positions on the rail, the extent of the change in the contact resistance with varying contact external load differs. In the relatively smooth areas of the rail surface with lower roughness, which are located in the latter half, the change in the contact resistance with contact external load is significantly smaller than that in the first half.

The reason for the different extents of contact resistance variation under the same change in contact external load but with different roughness is as follows: in the first half of the rail, the rail wear surface exhibits some delamination of the deposited layers, pores, and cracks, as well as mechanical wear, such as gouges. At this stage, there are gaps between the contact surfaces of the armature and rail, resulting in a smaller actual contact area. When the external load increases, the gaps between the armature and rail decrease, the number of contact asperities increases, and the increase in external load causes a significant change in the actual contact area. Therefore, the static contact resistance between the armature and rail is sensitive to the external load changes in this region. In the latter part of the rail, the rail wear surface exhibits signs of transition ablation and surface ablation. The rail surface is relatively smooth, and at this point, the contact between the armature and rail is good, with a higher number of contact asperities. When the external load changes, the actual contact area does not change significantly, making the static contact resistance between the armature and rail less sensitive to external load changes.

Each experimental data point represents the mean of five repeated measurements, and the error bars indicate the standard deviation (SD). Figure 9b shows the calculated static contact resistance between the armature and rail at different positions along the rail using the contact resistance model. The trend of the contact resistance calculated by the model is consistent with the experimentally measured contact resistance, which demonstrates the accuracy of the model.

Table 4 lists the errors between the experimentally measured and model-calculated contact resistance values. Most points have small errors, as low as 0.235%. Some points have relatively large errors of up to 9.807%. However, most errors are controlled within 10%, indicating that the model has relatively good stability and reliability for predicting the results.

## 4. Conclusions

Through conducting electromagnetic launch experiments, this study investigates the surface morphology, two-dimensional profile lines, and static contact resistance at different positions of the rail. Based on fractal theory, a contact resistance model that considers the actual contact area and external load is established. Using the height data of the two-dimensional profile lines of the rail surface, fractal dimension *D* and characteristic scale coefficient *G* are calculated and substituted into the contact resistance model to compute the contact resistance. The following conclusions can be drawn:

(1) The front half of the rail used in the experiment is primarily affected by mechanical wear, whereas the latter half of the rail is dominated by electrical wear. Along the armature launch direction, the fluctuation range of the 2D profile of the rail surface becomes smoother and the surface roughness decreases, with the smallest fluctuation occurring at position P6.
(2) Under experimental conditions, the contact resistance calculated using fractal theory shows better agreement with the experimental measurements than the GW model, demonstrating smaller errors. However, its applicability requires further verification under real engineering conditions.
(3) Under experimental conditions, the greater the roughness, the more sensitive is the contact resistance to changes in external load; the smaller the roughness, the lower is the sensitivity. When the surface roughness is relatively high, the actual number of contacting asperities under the same external load is relatively low. As the external load increases, the number of contacting asperities on the rougher surface increases more significantly than that on a smoother surface. Therefore, the contact resistance of surfaces with higher roughness is more sensitive to variations in the external load.

A static contact resistance model between the armature and rail is established by incorporating surface topography parameters, and the influence of wear on the contact resistance is further investigated. In future work, rail coating techniques will be considered to reduce the contact resistance and extend the rail lifespan. Additionally, current and velocity parameters will be incorporated to develop a dynamic contact resistance model.

## Figures and Tables

**Table 1:** Chemical composition of the alloy steel.

C	Si	Mn	P	S	Cu	Ni	Cr
0.17%	0.23%	1.53%	0.013%	0.008%	0.008%	0.004%	0.22%

**Table 2:** Material properties of armature and rail.

	Mass Density (g/cm^3^)	Elastic Modulus (GPa)	Poisson’s Ratio	Conductivity (S/m)
rail	7.85	205	0.28	9.6 × 10^6^
armature	2.8	72	0.30	3.8 × 10^7^

**Table 3:** The contact resistance values obtained from the GW model.

*F* (kN)	*R* at P1 (mΩ)	*R* at P2 (mΩ)	*R* at P3 (mΩ)	*R* at P4 (mΩ)	*R* at P5 (mΩ)	*R* at P6 (mΩ)
4	0.7068	0.8411	0.6238	0.5724	0.3723	0.2958
5	0.6561	0.7808	0.5782	0.5313	0.3456	0.2746
6	0.6174	0.7347	0.5441	0.5004	0.3253	0.2584
7	0.5865	0.6979	0.5168	0.4750	0.3089	0.2455
8	0.5609	0.6675	0.4943	0.4543	0.2955	0.2348
9	0.5393	0.6418	0.4753	0.4368	0.2841	0.2258

**Table 4:** The errors of the contact resistance model at different positions on the rail.

*F* (kN)	P1	P2	P3	P4	P5	P6
4	2.300%	2.049%	2.661%	3.448%	4.096%	3.432%
5	2.739%	0.483%	5.894%	1.409%	7.143%	3.025%
6	3.122%	0.248%	6.600%	4.378%	5.389%	9.807%
7	5.251%	0.412%	6.078%	4.484%	1.247%	0.417%
8	5.482%	0.451%	3.917%	2.393%	1.764%	0.909%
9	2.205%	1.597%	2.219%	1.173%	3.069%	0.235%

## Data Availability

The original contributions presented in this study are included in the article. Further inquiries can be directed to the corresponding author.
